# A novel RBF-based predictive tool for facial distraction surgery in growing children with syndromic craniosynostosis

**DOI:** 10.1007/s11548-019-02063-4

**Published:** 2019-10-31

**Authors:** F. Angullia, W. R. Fright, R. Richards, S. Schievano, A. D. Linney, D. J. Dunaway

**Affiliations:** 1 UCL Great Ormond Street Institute of Child Health, Paediatric Surgery Offices Room 160, 30 Guilford Street, London, WC1N 1EH UK; 2 Craniofacial Unit, Great Ormond Street Hospital for Children, Great Ormond Street, London, WC1N 3JH UK; 3 ARANZ Medical Ltd., 47 Hereford Street, Level 1, Christchurch Central, Christchurch, 8013 New Zealand; 4 Medical Physicist, 47 Westcott Road, London, SE17 3QY UK; 5 Cardiorespiratory Unit, Great Ormond Street Hospital for Children, Great Ormond Street, London, WC1N 3JH UK; 6 UCL Ear Institute, 332 Gray’s Inn Road, London, WC1X 8EE UK

**Keywords:** Radial basis function, Craniosynostosis, Facial distraction, Landmarks, Prediction, Surgical model

## Abstract

**Purpose:**

Predicting changes in face shape from corrective surgery is challenging in growing children with syndromic craniosynostosis. A prediction tool mimicking composite bone and skin movement during facial distraction would be useful for surgical audit and planning. To model surgery, we used a radial basis function (RBF) that is smooth and continuous throughout space whilst corresponding to measured distraction at landmarks. Our aim is to showcase the pipeline for a novel landmark-based, RBF-driven simulation for facial distraction surgery in children.

**Methods:**

An individual’s dataset comprised of manually placed skin and bone landmarks on operated and unoperated regions. Surgical warps were produced for ‘older’ monobloc, ‘older’ bipartition and ‘younger’ bipartition groups by applying a weighted least-squares RBF fitted to the average landmarks and change vectors. A ‘normalisation’ warp, from fitting an RBF to craniometric landmark differences from the average, was applied to each dataset before the surgical warp. The normalisation was finally reversed to obtain the individual prediction. Predictions were compared to actual post-operative outcomes.

**Results:**

The averaged change vectors for all groups showed skin and bone movements characteristic of the operations. Normalisation for shape–size removed individual asymmetry, size and proportion differences but retained typical pre-operative shape features. The surgical warps removed the average syndromic features. Reversing the normalisation reintroduced the individual’s variation into the prediction. The mid-facial regions were well predicted for all groups. Forehead and brow regions were less well predicted.

**Conclusions:**

Our novel, landmark-based, weighted RBF can predict the outcome for facial distraction in younger and older children with a variety of head and face shapes. It can replicate the surgical reality of composite bone and skin movement jointly in one model. The potential applications include audit of existing patient outcomes, and predicting outcome for new patients to aid surgical planning.

## Introduction

Surgical correction of facial deformity from syndromic craniosynostosis in growing children is a complex and challenging task. Surgeons operate on abnormally shaped and malpositioned facial bones to modify the contours of the overlying soft tissues. Surgical planning has to account for relative movements of facial bone and skin which are difficult to delineate and predict. Any strategy which attempts to predict changes from such facial surgery must incorporate both bone and soft tissue movements in a realistic way that mimics the surgical situation.

Syndromic craniosynostosis affects both facial bone and the overlying soft tissues. Children diagnosed with syndromic craniosynostosis such as Apert and Crouzon syndromes have characteristic deformities that affect facial appearance and function. Affected individuals typically have shallow, laterally rotated orbits, a mid-face which is retruded and abnormally shaped, dental malocclusion and an abnormal forehead contour [1]. Cranial proportions are turricephalic (‘tall’) and brachycephalic (antero-posteriorly ‘short’). There is more laxity in the facial soft tissues.

**Fig. 1:**
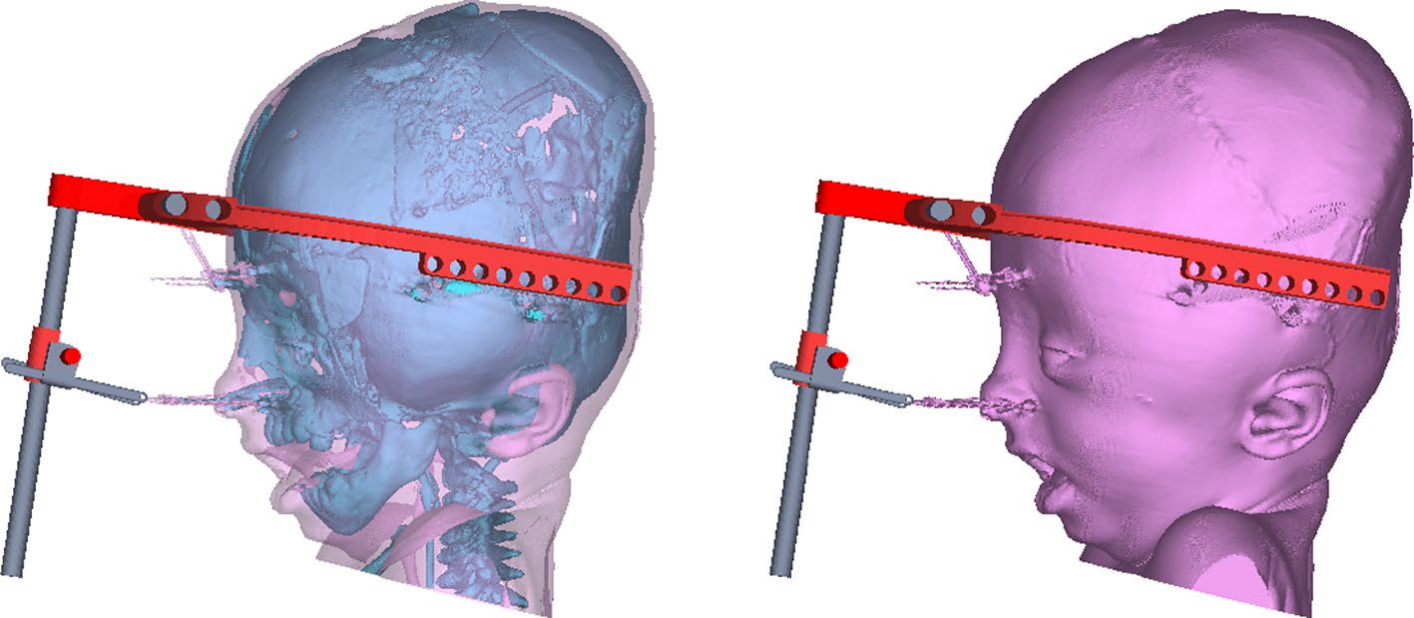
Position of the RED frame on a younger patient undergoing a monobloc facial distraction procedure (bone and soft tissue views)

Monobloc facial distraction (‘monobloc’) and its bipartition counterpart (‘bipartition’) are two surgical interventions used to correct such facial deformity. In the monobloc operation, bone cuts are made to free the forehead, orbits and mid-face from the cranium and skull base, followed by the application of a rigid external distractor (RED) device to the bone segments (Fig. 1). The ‘bipartition’ is likewise but includes the added steps of bony resection to medialise the orbits, and a midline osteotomy of the mid-face before distraction [2].

Gradual distraction of the bone segments is then performed over the period of a few weeks to slowly advance the forehead and mid-face ‘forward’, with the aim of creating a more acceptable and functional face shape. Distraction is complete when the soft tissue face shape is favourably judged by the surgeons. To date, there is no more-objective means of judging surgical outcome but relies solely on the surgeons’ perspective. This calls for a surgical model that could serve as a tool to guide the planning and progress of such procedures.

Abnormal facial bone and soft tissue shape require thoughtful and careful characterisation by an experienced surgeon in order for surgical prediction modelling to work. Any landmarks used must capture the relevant shape features of both skin and bone surface and be jointly incorporated into a mathematical function that is fit for purpose. This function should, in essence, replicate the smooth distraction process of the advancing bone stretching the overlying facial skin and be able to continuously model both operated and unoperated regions. It must also be versatile enough to model surgical change on faces of different sizes and asymmetry. A radial basis function (RBF) is one such candidate for the modelling of facial distraction surgery.

RBFs have been used to model shape from geological and environmental data, and in medical imaging in conjunction with laser scanning [3]. These functions have the feature of modelling data points in the ‘least wiggly’ format where a smooth modelling of shape is achieved.

Prediction of surgical outcome for facial distraction will be valuable for the audit and planning of facial distraction procedures, especially those which involve growing children with changing face shapes.

Firstly, the predictive tool will simulate the typical operation of its kind which can then be compared to the actual outcome. This can help the surgeon reflect on current practice and inform the plan of subsequent procedures.

Secondly, it allows the surgeon to compare the outcome of different procedures on individual patients and guide the choice of procedure that will produce the most acceptable and functional face shape.

Thirdly, it provides the opportunity for the surgeon to experiment on alternative techniques which may give a more favourable outcome in advance.

## Aim

Our aim for this study is to showcase the pipeline for a novel landmark-based, RBF-driven simulation for facial distraction surgery in growing children.

## Method

### Cohort selection and scan preparation

Three cohorts of children were selected for the study.

The older ‘monobloc’ group comprised of Crouzon patients (*n* = 20) and ‘bipartition’ group comprised of Apert patients (*n* = 16). The age range of the ‘older’ groups was from 7 to 21 years of age. The younger group consisted of Apert patients (*n* = 5) who had undergone monobloc facial bipartition surgery. The age range of the ‘younger’ group was 1–6 years of age.

All subjects had pre- and post-operative high-resolution 3-dimensional CT scans of the full head and face. The interstice spacing of the scans was 1 mm.

Segmentation of craniofacial bone (Hounsfield, 239) and skin (Hounsfield, − 224) iso-surfaces was performed from the DICOM format of the latest pre-operative and earliest post-operative scans for each subject.

### Landmarking

Landmarks were placed on every pre- and post-operative skin and bone iso-surface.

The landmark sets were designed and manually placed by the same surgeon to optimise surface coverage over regions of the face advanced by the operation. They describe anatomical features of facial bone and the overlying skin, as well as their topographical shape features.

**Fig. 2:**
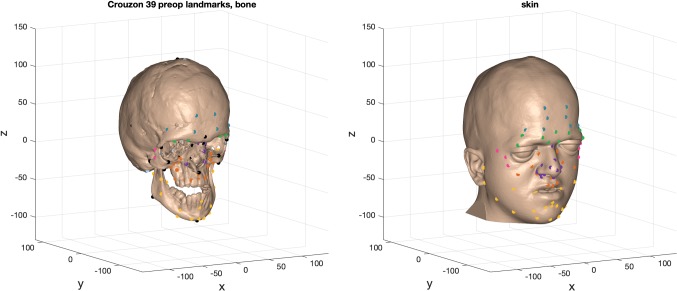
Example of bone and skin landmarks used to characterise the pre- and post-operative iso-surfaces for an individual patient. Black landmarks on the bone surface belong to the craniometric set

Seventy-eight skin landmarks were placed on all pre- and post-operative facial skin iso-surfaces. Seventy-eight bone landmarks [4] on the ‘moving’ (operated) bone iso-surfaces (Fig. 2). ‘Static’ (unoperated) regions of the craniofacial skeleton were constrained by placement of bone landmarks on the calvarium and skull base (Fig. 2).

### Reliability

To evaluate intra-operator reliability of landmark placement, a random bone and skin data set was chosen from each of the three cohorts and landmarked with skin and bone landmarks 10 times at different sittings by the same landmarker.

The standard deviation (SD) from the mean position of each landmark was calculated. The unpaired t test with equal variances with a two-tailed significance was used to assess differences in mean scores in all measurements. A value of *p* < 0.05 was defined as significant.

### Alignment

All skin and bone iso-surfaces with landmarks (‘patient data set’) were aligned in a novel, standardised 3-dimensional (3D) reference frame.

**Fig. 3:**
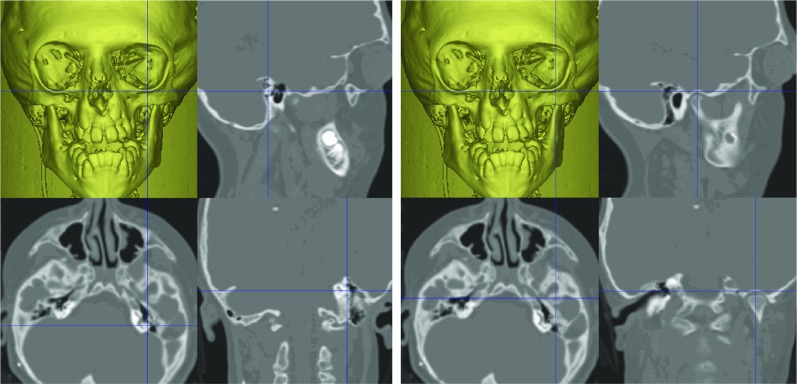
Technique for placement of vestibular (left) and glenoid fossa (right) landmarks

**Fig. 4:**
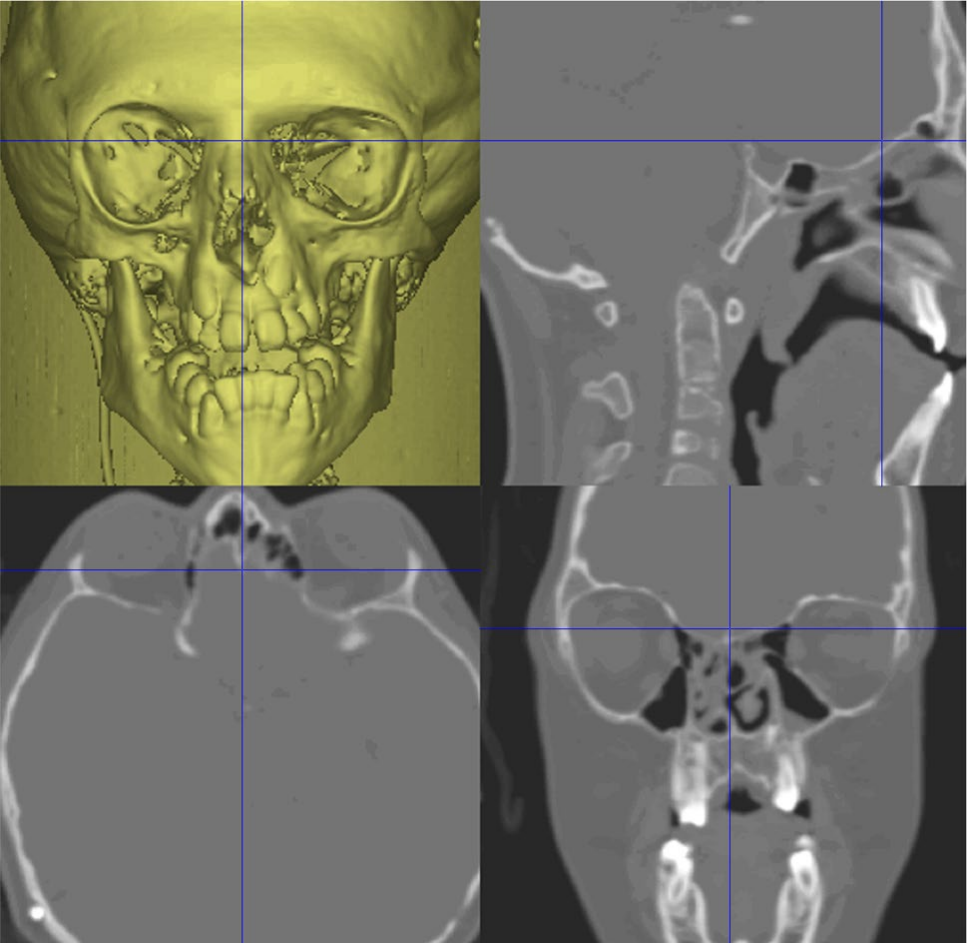
Technique for placement of the crista galli landmark

Landmarks used to define the reference frame were located in ‘static’ (unoperated) regions of the craniofacial skeleton (Table 1). The vestibular, glenoid fossae and crista galli anatomical points on the skull base were used to construct the reference frame (Figs. 3, 4).

**Table 1:** Reference frame landmarks

1	Right lateral semicircular canal (lateral)
2	Left lateral semicircular canal (lateral)
3	Right lateral semicircular canal (posterior)
4	Left lateral semicircular canal (posterior)
5	Right glenoid fossa
6	Left glenoid fossa
7	Crista galli

A best-fit plane was developed from the vestibular and glenoid fossae landmarks which were subsequently projected onto the plane.

A line was drawn through most-lateral vestibular landmarks (landmarks 1 and 2; Table 1). This was translated onto the *x*-axis. The midpoint of the landmarks was then translated onto the origin.

A best-fit plane (landmarks 1–6; Table 1) was then rotated about the *x*-axis to be co-planar with the horizontal *x*–*y* plane (Fig. 4).

**Fig. 5:**
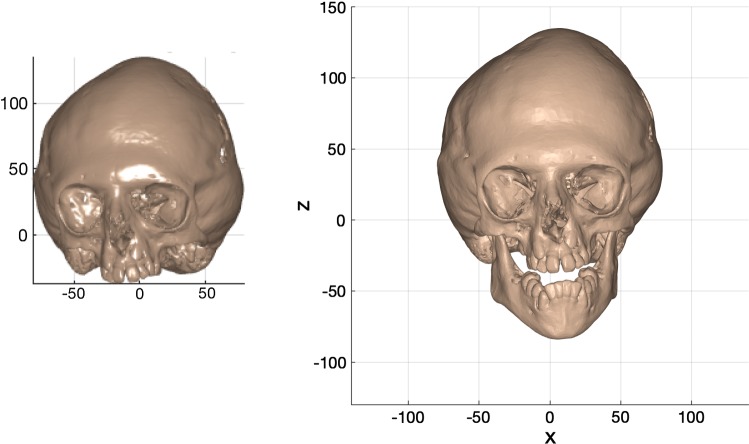
Left and right images are of the same pre-operative bone iso-surface and co-located in the same coordinate system (scale in mm). The bone surface on the left is co-planar with the *x*-axis but before rotation to coincide with the crista galli point. The bone surface on the right is after rotation (co-planar with *y*–*z* plane)

Following this, the landmarks (landmarks 1–6; Table 1) were rotated about the *z*-axis to bring the crista galli landmark (landmark 7; Table 1) onto the anterior *y*–*z* plane (Fig. 5).

### Surgical model

Three surgical models (‘warps’) were made: the ‘monobloc’, ‘bipartition’ and ‘younger bipartition’ models.

**Fig. 6:**
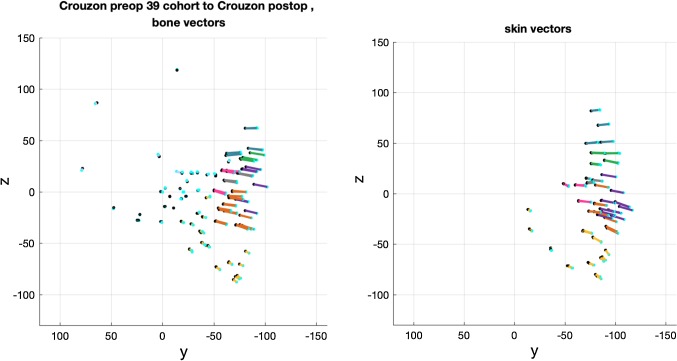
The averaged change vectors used to build the ‘surgical warp’ applied to an individual patient

The average locations of each bone and skin landmark were computed for both pre- and post-operative patient datasets. The change vectors between corresponding pre- and post-operative landmarks defined the change in shape due to surgery in operated regions of the head and face. Change vectors between corresponding landmarks on the unoperated cranium and skull base were also computed in the model to achieve a smooth and continuous model of surgical movement (Fig. 6).

A least-squares RBF, that describes a nonlinear change in shape, was fitted to the change vectors at the averaged pre-operative landmarks to produce the ‘surgical warp’. If the ‘surgical warp’ was required to exactly match the vectors and averaged landmarks, it could become quite ‘wiggly’ and physiologically unrealistic. Therefore, the balance between smoothness and fidelity was determined by the variance of the averaged landmarks. At landmarks with a high variance, the requirement for fidelity of the RBF was reduced, in favour of smoothness of the function. Thus, the resulting ‘surgical warp’ models craniofacial shape change between pre- and post-operative states in a physiologically and surgically realistic manner.

Surgical warps for the ‘monobloc’, ‘bipartition’ and ‘younger bipartition’ groups were produced with *n *− 1 of the relevant patient datasets.

### Normalisation

All patient datasets were ‘normalised’ for size–shape before application of the surgical warps.

Each pre-operative patient dataset was ‘normalised for shape–size by the application of another nonlinear, weighted, least-squares RBF, in the same manner as the surgical model.

The data for this warp were the vector change in locations of 36 craniometric landmarks, moving from the individual’s locations towards the cohort average. These were chosen to define craniofacial proportions relevant to the disease processes (Fig. 2).

As before, the RBF was weighted to the variance in the averaged craniometric landmarks. By applying it to an individual’s complete dataset, the individual variation in shape and size was accounted for before a surgical warp was applied to the patient datasets.

### Application of the surgical model

Post-operative predictions were made for the three cohort groups.

First, the normalisation warp was applied to an individual’s dataset so that the landmarks closely correspond to the average landmarks for that cohort. Then, the pre-computed surgical warp was applied to the normalised individual dataset. A normalised prediction was produced for that individual. Finally, the normalisation warp was applied in reverse to restore the individual’s shape and size to the normalised prediction.

### Evaluation of predictions

The resultant predictions were visualised in three modes; (1) change vector plots, (2) change overlays and (3) signed-difference function maps. Predictions were colour-coded according to the signed-distance deviation from the closest point on the actual post-operative outcome.

## Results

### Intra-operator reliability

All bone landmarks on the facial skeleton and skull base were within an SD of 2 mm. The calvarial (skull) bone and skin landmarks were within an SD of 3 mm. This was consistent across all three data sets.

Landmarks with SD < 1 mm are considered of ‘high’ accuracy (*p* < 0.01). Those measured between 1 and 2 mm SD were considered ‘accurate’ (*p* < 0.05), and those between 2 and 3 mm SD were considered ‘less-accurate’ (*p* < 0.07). All landmarks found to be < 1 mm SD were ‘anatomical’ landmarks, and those > 1 mm SD were based on surface curvature.

82.1% (*n* = 64) of facial skeleton and skull base landmarks were within 1 mm SD. The remaining 17.9% (*n* = 14) were between 1 and 2 mm SD (bone forehead landmarks). 79.5% (*n* = 62) of skin landmarks were within 1 mm SD, 15.4% (*n* = 12) were between 1 and 2 mm SD (cheeks and forehead landmarks), and 5.1% (*n* = 4) were between 2 and 3 mm SD (forehead landmarks). All seven calvarial bone landmarks were found to be between 1 and 3 mm SD, with the greatest variation from landmarks on the top, sides and back of the skull.

### Alignment of cohort

**Fig. 7:**
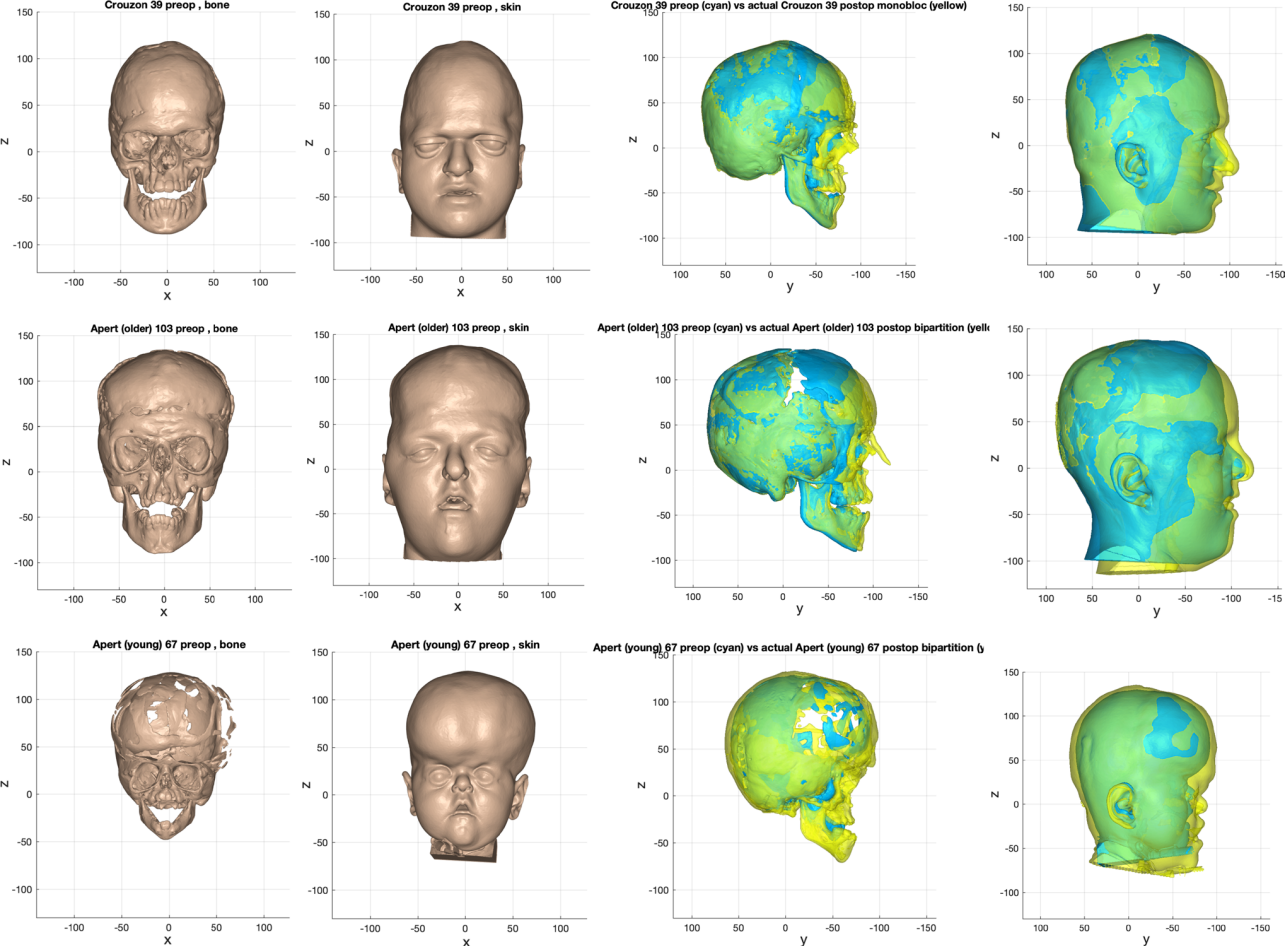
Pre- and post-operative bone and skin iso-surfaces in alignment for a patient from each cohort. Top row: example from the monobloc group; middle row: example from the bipartition group; bottom row: example from the younger bipartition group (scale in mm)

Both pre- and post-operative skin and bone iso-surfaces were localised in an acceptable ‘frontal’ position by the reference frame. Lateral rotation of the heads was eliminated (Fig. 7). There was no elevation or depression of the heads co-located in the same coordinate system (Fig. 7). This was observed for both Crouzon and Apert iso-surfaces.

Co-location of the pre- and post-operative iso-surfaces shows close congruence of unoperated regions, allowing for operated regions to be clearly defined. Translation was minimised between vastly different pre- and post-operative shapes. (Fig 7)

### Surgical models

**Fig. 8:**
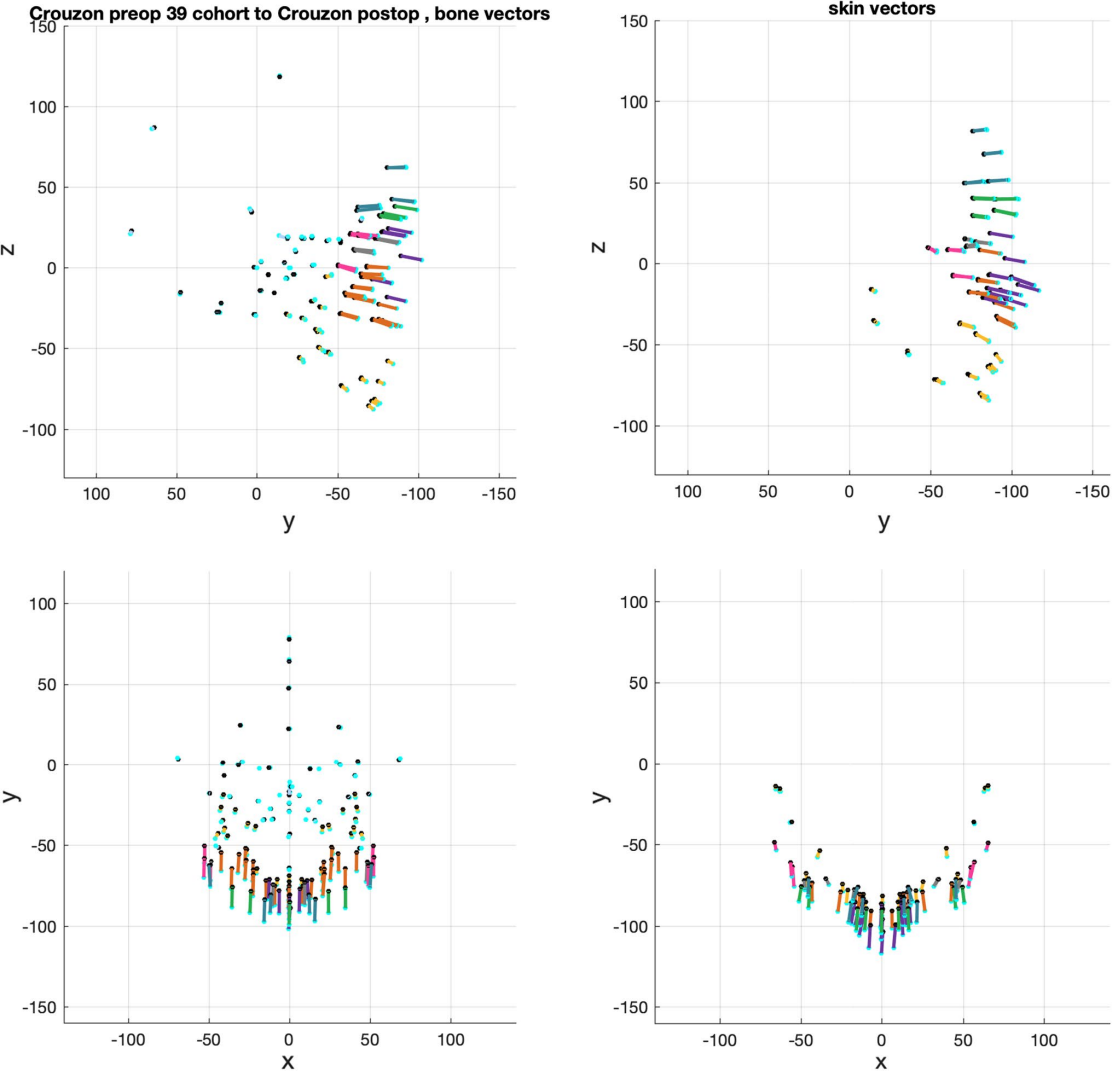
Averaged vectors that made up the surgical warp for the ‘older’ monobloc group as applied to an individual patient. Top row: profile view (*y*-plane); bottom row: bird’s eye view (*x*-plane)

**Fig. 9:**
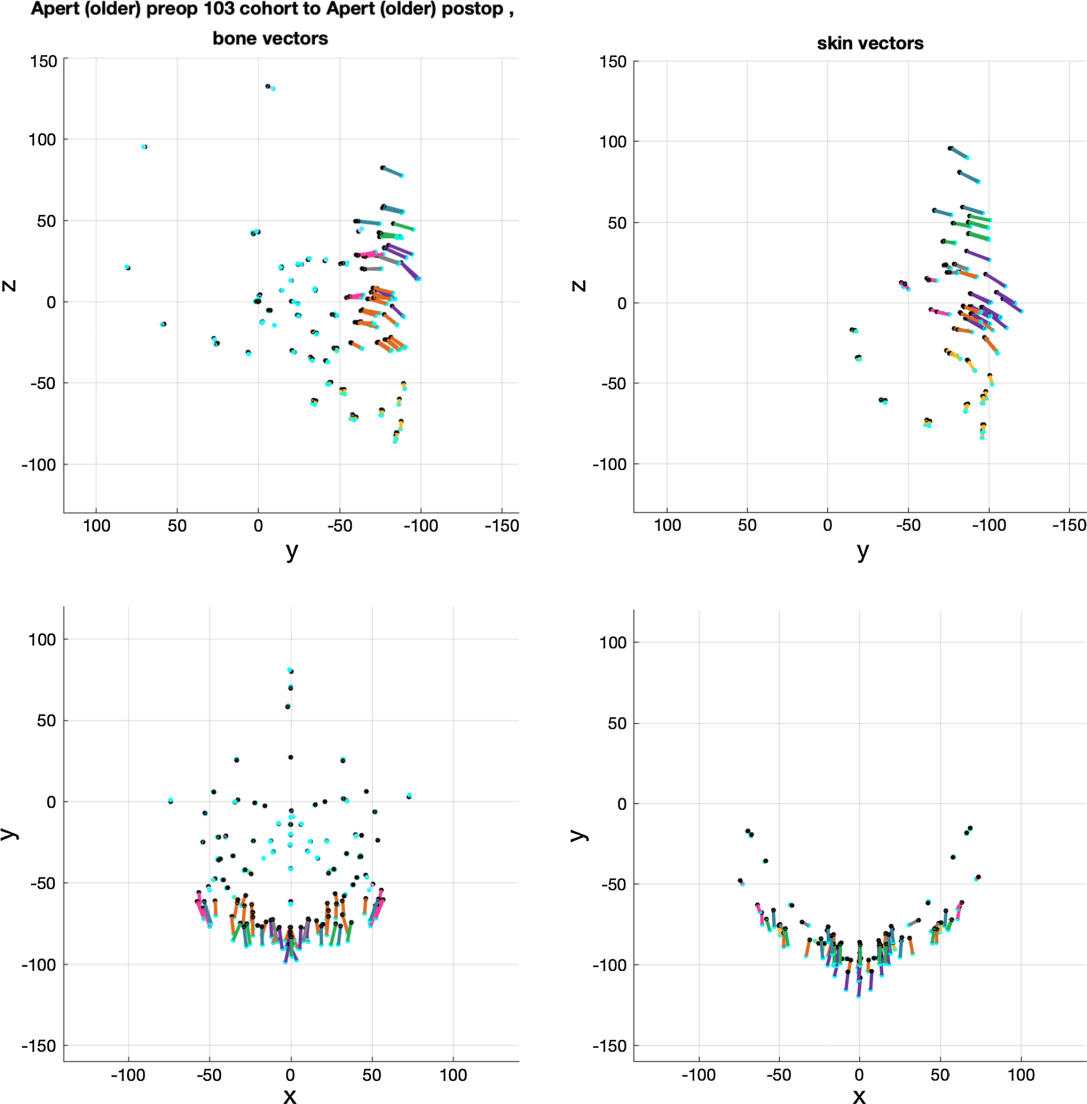
Averaged vectors that made up the surgical warp for the ‘older’ bipartition group as applied to an individual patient. Top row: profile view (*y*-plane); bottom row: bird’s eye view (*x*-plane)

**Fig. 10:**
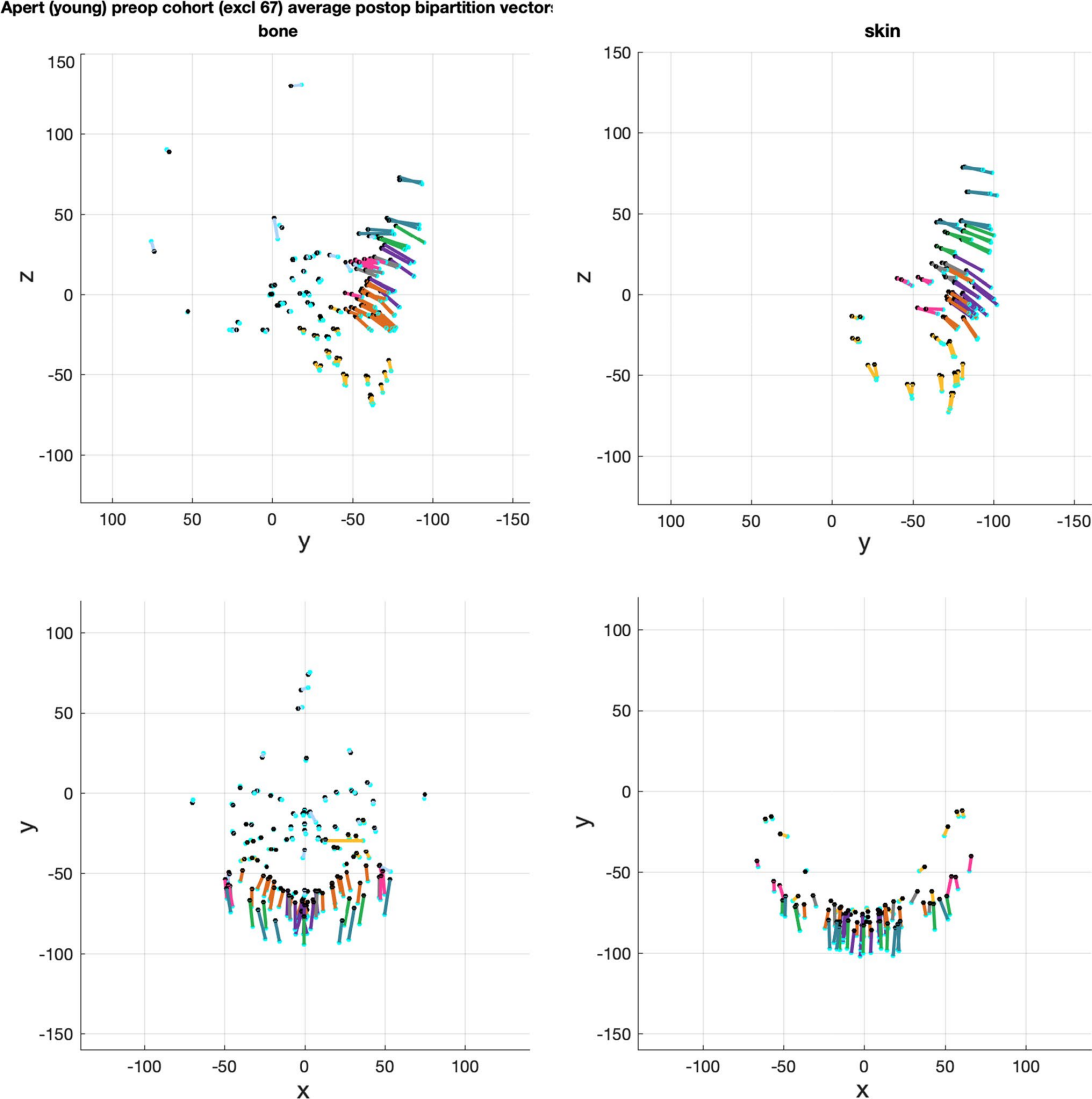
Averaged vectors that made up the surgical warp for the ‘younger’ bipartition group as applied to an individual patient. Top row: profile view (*y*-plane); bottom row: bird’s eye view (*x*-plane)

The averaged change vectors between pre- and post-operative iso-surfaces in the surgical warps are shown in Figs. 8, 9 and 10. Vectors are colour-coded according to the corresponding skin and bone regions they represent for ease of visualisation (Table 2). The averaged vectors comprise of the patient data sets in each cohort excluding the patient on which the warp is applied (*n *− 1 datasets). All vectors are plotted in millimetres (mm).

**Table 2:** Colour codes for vectors

Teal	Forehead
Green	Brow
Grey	Ocular
Purple	Nasal
Orange	Maxillary
Pink	Zygomatic
Yellow	Mandibular
Pale blue	Calvarium and skull base

These are the change vectors (*n* = 19) that made up the monobloc surgical warp (Fig. 8). There was a forward advancement of the forehead (teal) and brow (green) regions. The mid-facial (purple and orange) and zygomatic (pink) regions were moved forward and downward by the operation. The unoperated mandible (yellow) rotated down in response to the advancing mid-face. Skin and bone vectors followed a similar trajectory in the i-plane. The calvarium and skull base (pale blue) vectors demonstrated minimal change.

There was forward advancement of all operated facial regions in the *x*-plane for bone. The skin vectors in the *x*-plane showed a similar advancement. There was a drift towards the midline in the zygomatic region and away from the midline at the lateral brow region of the skin vectors to reflect soft tissue augmentation in the monobloc.

These are the change vectors (*n* = 15) that made up the bipartition surgical warp (Fig. 9). There was a forward and downward advancement of all operated regions of the face. The downward trajectory of vectors for both bone and skin was more marked in the mid-facial region (purple and orange) compared to the monobloc vectors. There was a greater degree of inferior rotation of the unoperated mandible (yellow). Skin and bone vectors followed a similar trajectory in the *y*-plane.

A forward midline advancement at the forehead (teal) and brow (green) level was observed in the *x*-plane for both bone and skin. In contrast, there was widening of the para-midline bone in the anterior mid-facial region demonstrated by diverging vectors (purple and orange) in the *x*-plane. The nasal (purple) vectors for skin showed minimal divergence in the *x*-plane. The trajectory of the zygomatic (pink) and lateral maxillary (orange) vectors demonstrated a narrowing of the mid-face more posteriorly in the *x*-plane with advancement.

Medialisation of the orbits was demonstrated by the movement of ocular (grey) vectors towards the midline in the *x*-plane for bone and skin. Likewise, for the calvarial and skull base vectors (pale blue) in the monobloc, there was minimal change for the bipartition.

These are the change vectors (*n* = 3) that made up the ‘younger’ bipartition surgical warp (Fig. 10). There was a greater degree of forward and downward advancement of all operated regions of the face compared with the ‘older’ bipartition group. The change vectors were of a larger magnitude in keeping with the surgical plan. Skin and bone vectors followed a similar trajectory in the *y*-plane.

Vectors in unoperated regions (pale blue) captured the change in shape and size between the pre- and post-operative iso-surfaces, demonstrating remodelling of the young skull.

### Normalisation

**Fig. 11:**
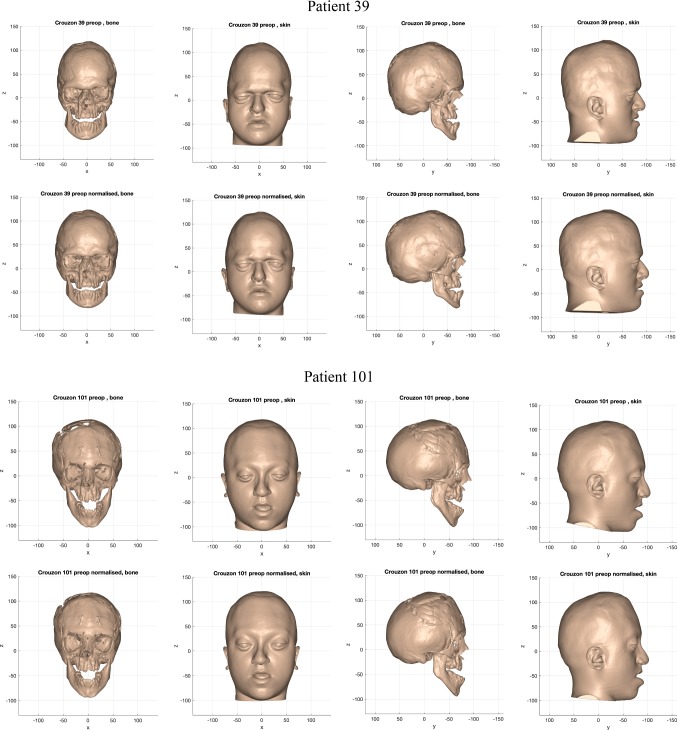
Example of the bone and skin outcome for the normalisation warp applied to 2 ‘older’ monobloc patients. Top row: pre-operative iso-surfaces in frontal and profile views before normalisation; bottom row: iso-surfaces in frontal and profile views after normalisation (scale in mm)

**Fig. 12:**
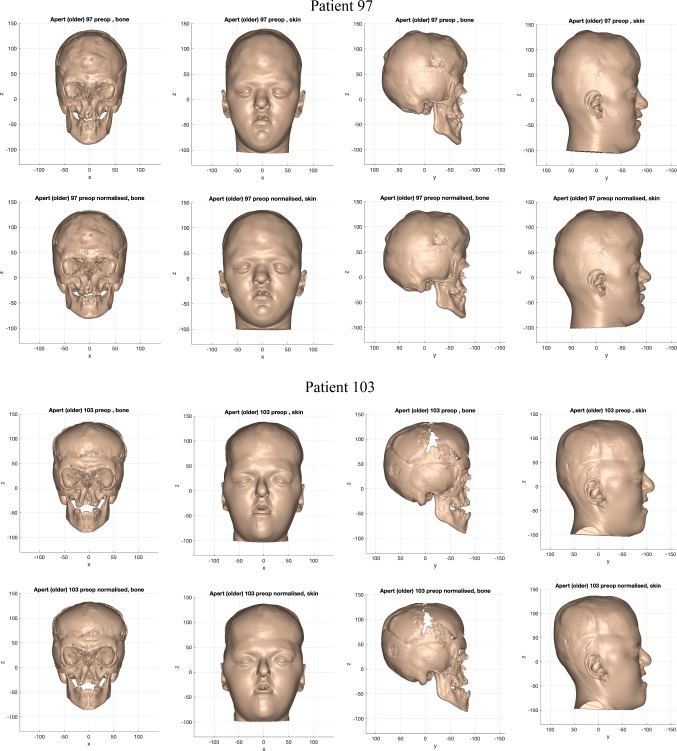
Example of the bone and skin outcome for the normalisation warp applied to 2 ‘older’ bipartition patients. Top row: pre-operative iso-surfaces in frontal and profile views before normalisation; bottom row: iso-surfaces in frontal and profile views after normalisation (scale in mm)

**Fig. 13:**
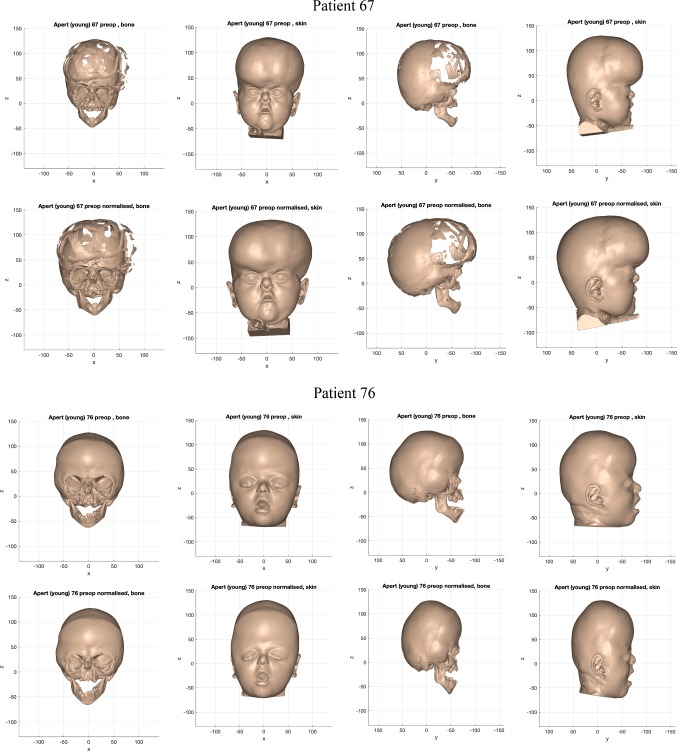
Example of the bone and skin outcome for the normalisation warp applied to 2 ‘younger’ bipartition patients. Top row: pre-operative iso-surfaces in frontal and profile views before normalisation; bottom row: iso-surfaces in frontal and profile views after normalisation (scale in mm)

The normalisation warp removed asymmetric features, size differences and variations of facial proportion from individuals in each cohort. Figures 11, 12 and 13 shows examples of the resultant normalised iso-surfaces from each cohort for bone and skin.

Taller heads were made shorter by the normalisation warp (Patient 39). Calvarial and facial asymmetry was removed. A reduction in width was observed in the wider heads and faces (Patient 101).

Similar results were obtained in the ‘older’ bipartition Apert group. Milder syndromic features such as downward-sloping canthi (Patient 97 skin) and laterally rotated orbits (Patient 97 bone) were emphasised by the normalisation warp. Conversely, the equivalent more-severe syndromic features were rendered less severe by the normalisation warp (Patient 103 skin and bone).

For the younger Apert bipartition group, where discrepancies of size are more marked, smaller heads were made bigger (Patient 67) and bigger heads were reduced in size (Patient 76) by the normalisation warp.

Overall, the normalisation warp removed shape–size features from individuals but retained features typical of the pre-operative syndromic state.

### Prediction process

**Fig. 14:**
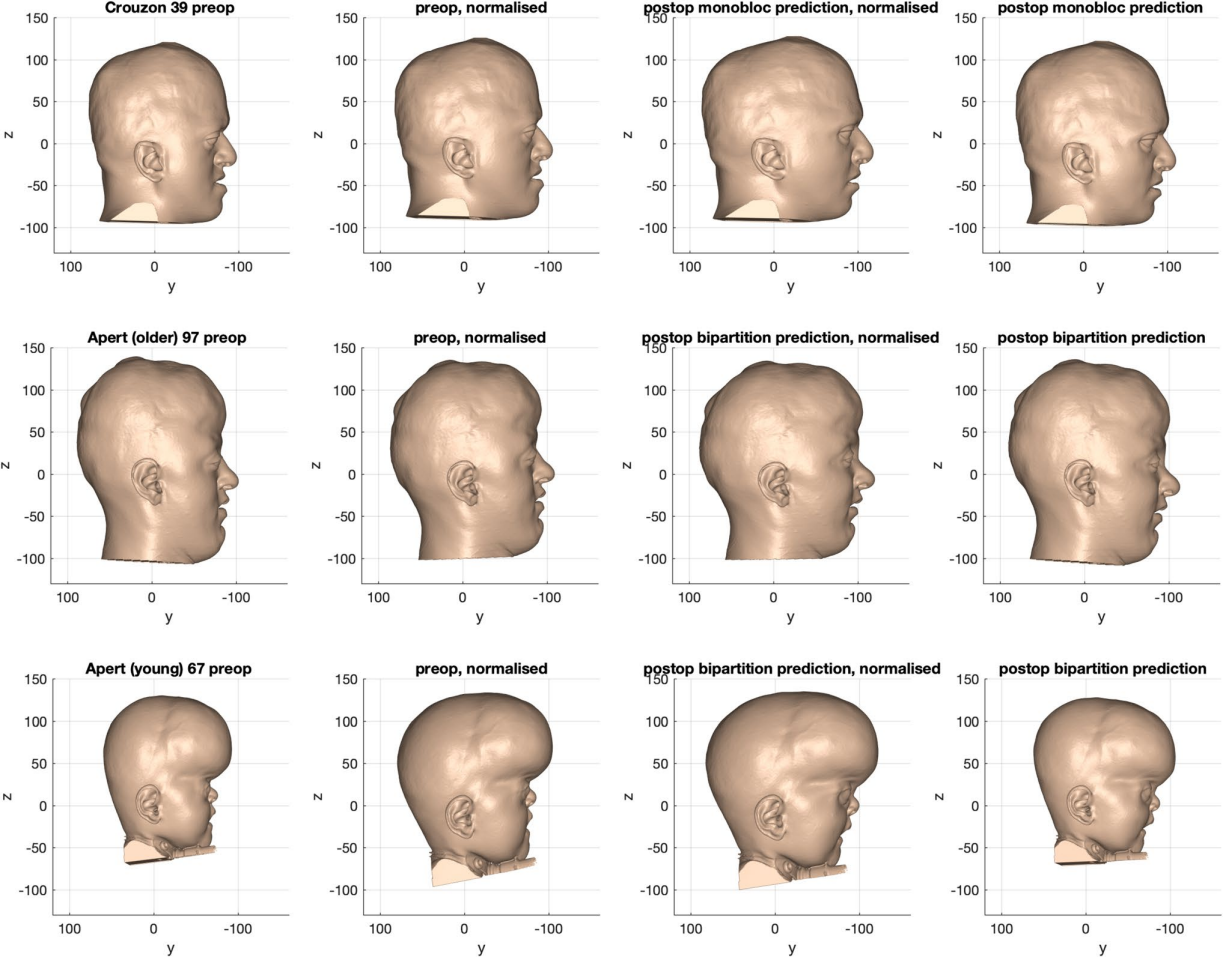
Example of the outcome of the prediction process for monobloc (top row), bipartition (middle row) and ‘younger’ bipartition (bottom row)

The outcome of the prediction process is shown in Fig. 14. The pre-operative state was normalised to exclude individual variation and disease severity. The surgical model was then applied to correct for the abnormal shape, eliminating typical shape features of the syndromes. Following that, the individual’s unique shape features were restored by reversing the normalisation warp on that dataset.

**Fig. 15:**
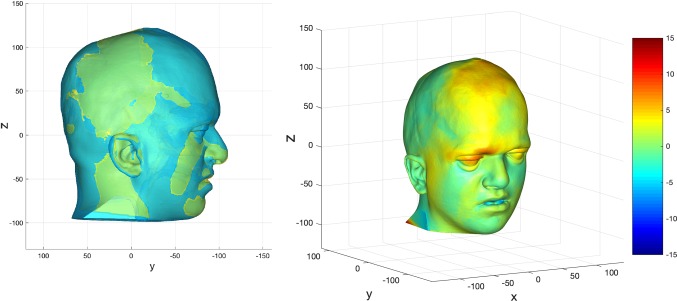
Prediction versus actual skin outcome comparison (left) for an individual monobloc patient. The difference between predicted and actual iso-surfaces is shown in a colour scale on the right (scales in mm)

**Fig. 16:**
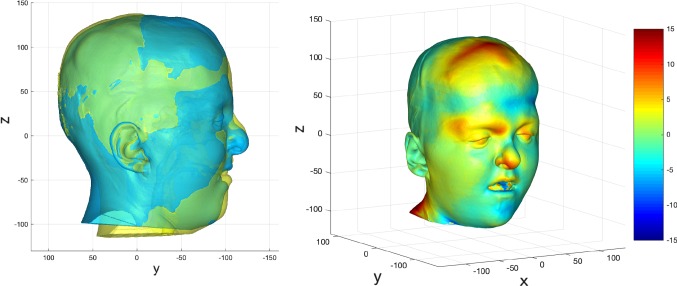
Prediction versus actual skin outcome comparison (left) for an individual bipartition patient. The difference between predicted and actual iso-surfaces is shown in a colour scale on the right (scales in mm)

**Fig. 17:**
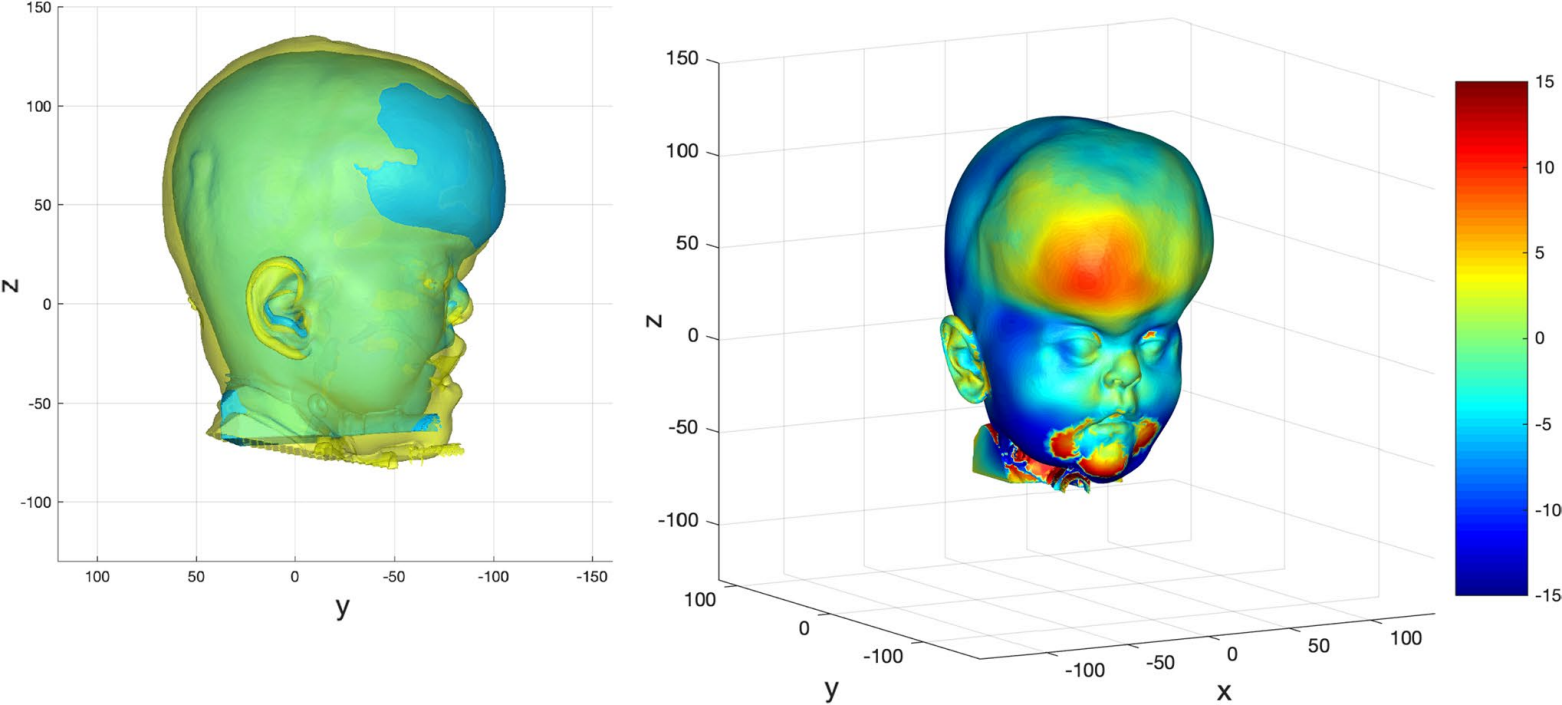
Prediction versus actual skin outcome comparison (left) for an individual ‘younger’ bipartition patient. The difference between predicted and actual iso-surfaces is shown in a colour scale on the right (scales in mm)

The predictions were compared to their actual post-operative datasets. Examples of these are shown in Figs. 15, 16 and 17.

The images on the left with two surfaces show the comparison between predicted (cyan) and actual (yellow) post-operative outcome. The colour difference map shows the signed-differences between predicted and actual post-operative skin iso-surface. A good match would show congruent curvature between blue (predicted) and yellow (actual) on the left, and a green colouration on the difference map scale on the right. All scales are in mm.

There is a good match between predicted and actual post-operative outcome in the mid-facial and lower facial regions for the monobloc group. The brow was over-predicted and the forehead less well predicted overall.

For the bipartition operation in older children, the predictions worked well in the mid-facial regions with a good predicted-actual contour match and a difference in surfaces of 0–2 mm. As with the monobloc group, the brow was over-predicted and the forehead outcome less well predicted. Mandibular position was predicted well in some cases but under-predicted in others (Fig. 17). The nasal regions were less well predicted in most individuals.

A good match between predicted and actual post-operative outcome was achieved for the mid-facial region. As with the older bipartition group, the forehead was less well predicted. Difference in size of the pre-and post-operative iso-surfaces rendered deficiencies in the lateral and lower facial prediction.

## Discussion

Our weighted nonlinear RBF fitted to landmarks and vectors which replicate facial distraction surgery is able to predict outcome in keeping with the surgical situation. It is a novel application of the RBF to modelling facial surgery landmarks that characterise the craniofacial skeleton and its overlying soft tissues well. The prediction process is versatile across a large variation of face shapes and sizes, as well as different surgical techniques.

A mathematical function that aims to model 3-dimensional surface data can only perform well if the data is well-represented enough to inform it. Strategic landmarks can be harnessed to characterise the operated facial regions, and unoperated regions to constrain the warps. They are particularly useful for less anatomically defined surfaces where there might be ‘loss of data’ such as bone gaps on the crown and remodelling at the forehead regions.

Manual landmarks come with an inherent human error which must be accounted for in the prediction process. The advantage of our prediction method is firstly, landmarks are placed by a single expert with a good intra-operator reliability. Predictions were better with less-reliable landmarks than with no landmarks over the calvarium and forehead regions.

Secondly, the RBF weighted to the variance of each averaged landmark so the resultant prediction mimics the smooth distraction process of bone stretching overlying skin without losing the fidelity of key anatomical landmarks like the lateral canthi which are surgically repositioned during the operations.

The third advantage is the application of the normalisation warp which acts to reduce the variance at averaged landmarks. This is important for data of high shape variation and also reduces effects of manual error in landmark placement.

And finally, the combined skin and bone landmark sets used in this prediction pipeline were paramount in jointly informing the RBF in one smooth and continuous model, without decoupling bone from skin movements.

The differences in face shape and size between individuals within the cohorts may be comparable to the difference surgery makes to an individual’s face shape. The effects of surgery are isolated from the effects of shape–size variation by decoupling the modelling of the surgical change and the normalisation for shape–size.

Inherent differences in shape within the cohorts also present a challenge for some methods of co-locating comparative scans, like iterative closest point (ICP)-based strategies [5]. Co-location of datasets using common internal anatomy which remains unaffected by surgery or disease overcomes this challenge. The novel reference frame we developed based on unaffected and unoperated structures removes the reliance on surface-matching methods in a dataset with a high shape variability.

Other co-locating strategies such as those used routinely for measurement of treatment outcomes in orthognathic surgery rely on facial landmarks such as the nasion, A and B points [6]; all of which move with facial distraction surgery. Instead, by relying on intrinsic balance organs to define the ‘horizontal’ adjusted to the anterior skull base in our co-location strategy, movement error from alignment landmarks is minimised when measuring change from facial distraction.

The components of the monobloc and bipartition facial distraction procedures that are common to all patients, regardless of individual face shape, were well-simulated by our prediction process. This is partly explained by the design and placement of the distractor device, and variation of surgical technique between patients.

Distraction at the orbital and mid-facial regions is best controlled by the distractor device where the tension wires are placed. It is not surprising that the predictions are most successful in these regions.

The advancement of the monobloc and bipartition segments was predicted well in the younger and older patients. Where there was no patient-specific surgical remodelling of bone, the post-operative mid-facial and orbital shapes were predicted well. Brow correction was less well predicted due to the higher degree of surgical remodelling and shape differences from metal implants. Foreheads were least well predicted as the ‘most-bespoke’ aspect of the operations.

Bespoke nasal bone grafts made nasal shapes less well predicted. Surgical remodelling of the forehead additionally reshapes a region where there are fewer landmarks to characterise. Therefore, our prediction process which relies on applying the common surgical technique between individuals is limited in predicting the individualised components of the operations.

The predictions demonstrated a descent of the mid-face that was more marked in the older bipartition Apert group than the monobloc Crouzon group. It is expected for gravity to have some influence on advancement by the RED. Perhaps the difference in downward drift lies in the stability of the facial bones during the distraction process. The bipartition is typically hinged in the upper mid-facial midline leaving the lower mid-facial segments free of each other. A less-rigid distracting segment may be the cause for that descent.

The downward auto-rotation of the mandible is a response to mid-facial distraction and descent. It is worse in the bipartition and disrupts surface difference mapping with the signed-distance function. This might account for the more marked surface differences in lower facial regions than otherwise shown in the predicted versus actual post-operative shape.

Improvement in less well predicted regions can be achieved by increasing the data representation with landmarks in those regions. Additional landmarks based on surface curvature [7] to constrain the top of the skull and the intracranial skull regions behind the forehead improved prediction in those regions.

Other types of RBF [8], such as the Hermite variety, are alternative solutions to improving accuracy of prediction without additional landmark data points. This would be a valuable next step in the refinement process of the surgical models.

## Conclusions

Our landmark-based, weighted RBF prediction process for simulating facial distraction surgery is novel, fit for purpose and able to model heads and faces of varying size and shape. This feature will be useful in any shape-changing procedure involving faces of growing children with 3D CT data, and also that of adults with a variety of face shapes.

It is a potentially useful audit tool for surgeons to assess how well their current surgical techniques are able to achieve desired outcome for complex conditions like syndromic craniosynostosis. The ability to predict the outcome of more than one surgical procedure gives surgeons the opportunity to simulate the alternative technique to their chosen surgical plan.

Our prediction process also opens up the possibility for patients to visualise what they might look like after surgery, before the actual procedure takes place. This can be a means of guiding the patient decision to undergo face shape-changing surgery, as well as the surgical plan thereof.

Where the prediction process is less good in simulating surgical change, further development in landmark-deficient areas such as the forehead may improve the model’s ability to predict more-bespoke aspects of the operations. Applying the predictive model to prospective patient data sets as part of the surgical planning process would test its robustness and usefulness in the clinical setting.
